# Medium-term results of Mini-arc for urinary stress incontinence in ambulatory patients under local anesthesia

**DOI:** 10.1590/S1677-5538.IBJU.2015.0655

**Published:** 2016

**Authors:** Almog Levi, Rasha Nasra, Inbar Ben Shachar, Naama Marcus Braun

**Affiliations:** 1 Department of Obstetrics and Gynecology, Ziv Medical Center, Faculty of Medicine, Bar-Ilan University, Safed, Israel

**Keywords:** Urinary Incontinence, Stress, Anesthesia, Local, Patient Satisfaction

## Abstract

**Objective:**

To evaluate the medium-term outcome and patient's satisfaction after Single-incision mini-sling (SIMS) procedure done under local anesthesia in ambulatory set up for patients with stress urinary incontinence (SUI).

**Materials and Methods:**

This is a retrospective cohort study, including all patients submitted to SIMS procedure for SUI with MiniArc (AMS, U.S.A) without concomitant surgery between January 2011 and March 2013. Patients were followed up during 12 months after surgery and once a year subsequently. Telephone interviews were conducted to evaluate patient satisfaction. Outcome masseurs included: SUI cure rate, urinary urge incontinence (UUI) cure rate in patients with mixed urinary incontinence (MUI), intra and post-operative complications and patient satisfaction.

**Results:**

Ninety-three patients were included with mean follow-up of 23 months. Fifty percent had MUI with predominant SUI. The cure rates of SUI (objective and subjective) were 89%. UUI was cured in 40% of patients. No major complications occur, neither voiding obstruction or groin pain. Telephone interviews conducted after 26 months on average revealed high satisfaction rate from the procedure (8.8 out of 10) and from the local anesthesia. Visual analog scale (VAS) rating was low during and after the procedure (2.38 and 2.69 respectively).

**Conclusions:**

The SIMS procedure is safe and highly effective for SUI and it can be performed successfully under local anesthesia in an ambulatory setup.

## Introduction

Stress urinary incontinence (SUI) affects the quality of life and wellbeing of approximately15% of the female population (1) and up to 35% of adult women worldwide (2). The treatment options for SUI include non-surgical options such as pelvic floor muscle training (PFMT) and surgical treatment. Surgery is generally more effective for severe SUI than PFMT is (3). The introduction of the mid-urethral sling (MUS) at the beginning of the 1990s by Petros and Ulstman (4) caused a complete shift from abdominal surgery, such as the Burch procedure, to the MUS procedures. Currently, MUS procedures are the most common and acceptable for SUI (5). Initially performed with retro-pubic approach, in the last ten years MUS procedures shifted toward the transobturator approach and toward single-incision mini-slings (SIMS), which help reduce complications of MUS such as hematomas, bladder perforation, groin pain, and obturator nerve capture (6).

Although the TVT-Secur (TVT-S, Ethicon, Summerville, USA), the first SIMS to be introduced, proved to be less successful than the retro-pubic and transobturator MUSs (7, 8), there are many other SIMSs that provide excellent results, as good as MUS, with lower complication rates (9–14). All MUS procedures can be performed under general, regional, epidural, or local anesthesia, but the common practice for the retro-pubic and transobturator sling procedures is still to perform them under general or regional anesthesia, followed by one-night hospitalization with Foley catheter. There is economic advantage to an ambulatory setup (15), and there are first reports of SIMS performed as an outpatient procedure (2, 16).

Some studies have examined the efficacy of MUS on mixed urinary incontinence (MUI) by monitoring the improvement in urgency and urgency urine incontinence (UUI) (17, 18). However, in some of these studies, patients had a concomitant pelvic organ prolapse (POP) repair; therefore, the improvement of symptoms could have been related to the prolapse repair. Furthermore, most MUS procedures included in these studies were retro-pubic or transobturator, therefore data regarding the efficacy of SIMS on MUI are lacking.

The aim of the current study was to evaluate the medium–term outcome and patient satisfaction after the MiniArc (AMS, U.S.A) procedure performed mainly under local anesthesia in an ambulatory setup in patients with SUI or MUI, without comorbid POP.

## Patients and Methods

This is a retrospective cohort study that included all the patients at Ziv Medical Center who underwent the SIMS procedure with MiniArc (AMS, U.S.A) without concomitant surgery between January 2011 and March 2013. The study was approved by the local ethical committee. Data were collected from medical records, including demographic parameters: age, body mass index (BMI), parity, medical history and previous gynecological surgeries, and pre-and post-operative evaluation. Preoperative consultation included a structured questionnaire for symptoms of SUI, urgency, urgency incontinence, and nocturia. The clinical examination included the Cough Stress Test (CST) with a full bladder in supine position and pelvic organ prolapse-quantitative (POP-Q) examination to rule out significant pelvic organ prolapse. SUI was determined based on positive CST, with obvious urethral hyper-mobility. MUI was determined based on obvious symptoms of SUI and UUI, absence of neurological disease, negative urinary culture, and positive CST. Urodynamic tests were not performed routinely in the presence of a predominant SUI in non-complicated patients. Patients were evaluated 6 weeks, 6 months, and 12 months after surgery, and once a year subsequently. Follow-up evaluation included symptoms of SUI, urgency, UUI, and nocturia. Post-operative examination included CST with full bladder and vaginal examination for mesh erosion or pain. Operative failure was defined as ongoing symptoms of SUI or positive CST.

Additionally, a telephone interview was conducted to evaluate patient satisfaction with the procedure performed under local anesthesia and with the overall process. It was conducted by a medical student, as part of his graduate degree, and he was blinded to the results. The interview included 7 questions, which were answered subjectively by the women. Experienced pain was evaluated using the visual analog scale (VAS).

The MiniArc procedure was performed during day-care hospitalization and under local anesthesia. In rare cases, general or spinal anesthesia was used upon patient request or based on a narrow vaginal access. Intravenous prophylaxis, Cefazolin 2gr, was given preoperatively. There was no routine Foley catheterization during or after the procedure. Local anesthesia included the injection of 2 Ampules of Lidocaine 1% to the para-urethral space, from the vagina towards the obturator foramen on both sides. The vaginal wall was opened 1cm under the urethra and the para-urethral fossa was opened on both sides towards the obturator foramen with blunt scissors until the ramus of the pubic bone was felt. The sling was placed on a delivery needle, which was then inserted through the incision and into the obturator foramen, into the obturator internus muscle. This was repeated with the second sling tip on the contralateral side. Sling tension was controlled by the operator. Once the sling was deposited, the delivery device was removed and the incision was closed. Women were discharged after spontaneous urination without urinary retention and residua.

Outcome measures included SUI cure rate in the entire group and in the MUI group, UUI cure rate in the MUI group, intra-and post-operative complications, and patient satisfaction.

## Results

**Figure 1:**
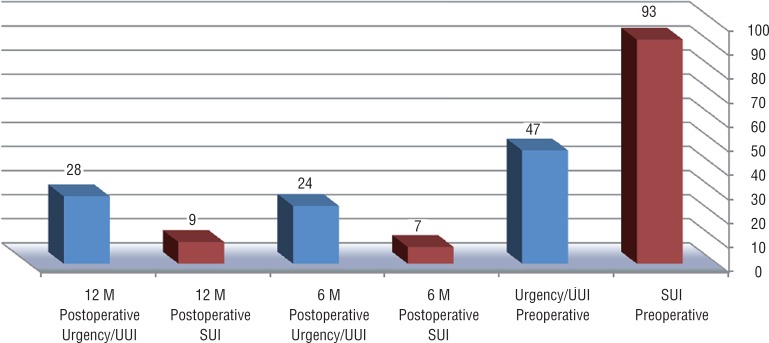
Stress urinary incontinence, urgency, and urgency urinary incontinence preoperatively, 6 months, and 1 year after MiniArc procedure. SUI – Stress Urinary Incontinence; UUI – Urgency Urinary Incontinence.

Ninety-three women who underwent placement of the MiniArc without POP repair during the study period were included. One surgeon (NMB) operated all cases. Mean patient age was 53 years (range 33–75), mean BMI was 28 (range 16.8–41.9), and mean parity was 3.3 (range 0–8). Mean follow-up at the clinic was 23 months (range 12–48). Forty-seven out of 93 patients (50%) had MUI with predominant component of SUI. Seventy-eight patients (84%) were operated under local anesthesia. One patient (1%) was switched from local to general anesthesia during the procedure because of pain. No intra-operative complications were reported, and the majority of patients (92%) were discharged after three hours on average. Three patients stayed overnight because of a transient urinary retention, which was resolved after 12h with the use of a Foley catheter. The overall cure rate of SUI (objective and subjective) was 89% (83 patients) (Figure-1). Of the remaining ten patients, two cases were considered as failure and required a second procedure. In both cases urodynamic test was performed prior to the second procedure to exclude other pathology. The second procedure used was retro-pubic MUS and was successful in both cases. Eight patients reported partial relief. There were no major long-term complications (Table-1).

**Table 1:** Long-term complications and treatment.

Complication N (%)	Detection	Treatment
Small mid-line erosion 2 (2%)	During first year	Removed under local anesthesia
Mesh penetrating the vagina (Unilateral) 2 (2%)	First follow-up	Mesh removed in OR^a^
Voiding dysfunction 1 (1%)	Immediate post-op^b^	Resolved spontaneously after 1 month
Groin pain – right due to hematoma 1 (1%)	Immediate post-op	Resolved spontaneously after 6 weeks
de novo UUI^c^ 2 (2%)	First follow-up	Successfully treated with anti-cholinergic drugs

**OR^a^** = Operation room; **PO^b^** post-operative; **UUI^c^** = Urgency urinary incontinence

Twenty-three out of 47 patients (49%) with MUI, experienced complete disappearance of urgency and UUI after 6 months of follow-up, decreasing to 19 out of 47 patients (40%) after 12 months, without further deterioration in the long term (Figure-1).

The telephone interview was conducted 26 months on average after the procedure. Eighty out of 93 women (86%) answered the questionnaire, and their responses are summarized in Table-2. There was an overall high satisfaction with the procedure (8.8 out of 10), and VAS ranking was low during and after the procedure (2.38 and 2.69 respectively). Most patients (90%) were happy with the local anesthesia, and only 10% would have preferred to undergo the procedure under general anesthesia because of pain.

**Table 2:** Results of the telephone survey.

	Average (range)	Range
1. What was the level of pain during surgery?	2.38 (0–10)	0–10 VAS
2. What was the level of pain after surgery?	2.69 (0–8)	0–10 VAS
3. How long did the pain last (in days)?	4.96 (0–90)	
4. How satisfied are you with the procedure?	8.82 (1–10) Solved 68 (85%)	0–10
5. Did surgery solve the problem?	Partial 10 (12.5%) Failed 2 (2.5%)	
6. Would you prefer a different kind of anesthesia for the operation you had?	No 72 (90%) Yes 8 (10%)	
7. Are you satisfied with the explanation about the procedure provided to you before the operation?	No 1 Yes 79	

## Discussion

Mid-urethral slings altered dramatically the treatment for SUI since they have been introduced by Ulmsten and Petros in 1995 (4). Complications of the retro-pubic MUS, such as vascular and bowel injuries, as well as bladder perforation and voiding dysfunction, led to the introduction of the transobturator MUS in order to decrease complication rates. Although the transobturator route reduced complications, it was associated with other complications such as vaginal penetration, de novo urgency, and persistent groin pain. Groin pain can be caused either by the passage of the mesh inside the obturator muscle or by contact with of the obturator nerve or even injury to it (6).

The aim of the SIMS procedure, first introduced in 2007 with the TVT-S, was to reduce MUS-related complications by obviating the need for the blind passage with trocars through the Retzius space or the transobturator foramen, and achieving the same success rate as other MUS

procedures. TVT-S did not stand up to expectations, and success rate was around 60%, as reported in many studies (19, 20). Other SIMSs appeared in the course of 2008 and thereafter, providing better results than the TVT-S did, and reducing complication rates (9–14). In 2011, TVT-S was still dominant in the market; therefore the meta-analyses that compared the transobturator and the retro-pubic MUS with SIMS included mainly studies involving TVT-S, and concluded that SIMSs are inferior to other MUSs (7). But recent meta-analyses that compare the results achieved with SIMS to those achieved with other MUS showed SIMSs to be highly promising, if studies with TVT-S are excluded from the sample (21). Therefore, we can conclude that not all SIMSs are the same, and that currently there are quite a few SIMS (MiniArc among them) that provide high success rate and few complications. However, more prospective randomize trials should be performed to compare between MUS and SIMS before final conclusion.

Performing the procedure under local anesthesia in an ambulatory setup has many advantages: significantly reducing the high cost of inpatient hospitalization and patient recovery (15), reducing anesthesia-related complications, and achieving high patient satisfaction with the procedure. In our study we found that patient satisfaction was very high (8.8 out of 10), and the majority of interviewers answered that they did not regret undergoing the procedure in this form. The process of administering local anesthesia involves certain difficulties, because the surgeon must rely on patient's cooperation. We assume that the lack of such cooperation was the reason for vaginal penetration in the first two cases. Careful examination at the end of the procedure can prevent this outcome. Although the procedure under local anesthesia can be technically somewhat more challenging, the present study demonstrated a high long-term success rate.

SUI is the most common form of urinary incontinence, occurring in pure or mixed forms in nearly 80% of women with incontinence (22). Patient history, symptoms, and physical examination (including positive CST) can identify most patients with a significant stress incontinence component. Urodynamic tests are controversial in cases of pure SUI, when urethral hypermobility is present. According to the studies by Nager and the Urinary Incontinence Treatment Network (23), a basic assessment for women with uncomplicated predominant SUI, who show stress incontinence on outpatient evaluation, is not inferior to a preoperative evaluation that also includes urodynamic testing. That routine urodynamic tests are unnecessary in relation to post-operative complications was also demonstrated by a recent meta-analysis, where the authors concluded that “In women undergoing primary surgery for SUI or stress-predominant MUI without voiding difficulties, urodynamics does not improve outcomes-as long as the women undergo careful office evaluation” (24). In the Ob/Gyn Department at Ziv Medical Center, urodynamic tests are not performed routinely in cases of predominant SUI without POP and before the primary operation. Although almost 50% of patients had MUI, all cases undergoing primary surgery had predominant SUI.

The telephone interview, conducted 26 months on average after the procedure, demonstrated a high satisfaction rate with the entire process and with the local anesthesia. Eighty-five percent reported complete cure, 12.5% partial cure, and only 2.5% reported that the procedure has failed. These numbers are somewhat different from the results in the clinic (89% complete cure), which may be explained by the nature of question 5 (Table-2), which asked whether the operation resolved the problem without addressing the SUI symptoms specifically, therefore women still suffering from UUI may have reported “partial relief.”

The present study has some limitations because of its retrospective nature, therefore interpretation of the data may be biased. Since there are no validated questionnaires in Hebrew regarding satisfaction, we used a built questionnaire which is a drawback. Moreover, the questionnaire includes questions about the pain experienced during and after surgery, the answers to which may be less than accurate, because of the time elapsed after surgery.

The strength of this study lies in the fact that it reports on a one-center experience, with procedures performed by the same surgeon, therefore there were no differences in surgical technique and protocol. All the patients in this group underwent surgery for SUI without concomitant prolaps repairs, therefore without any undesirable effect on functional or anatomical results. Finally, data collection from patient files was done by a medical student who was not involved in the surgeries. The telephone interview was conducted by an investigator who was blind to the data collection results, represented by a combination of questions about stress and urge incontinence.

## Conclusions

We found that the SIMS procedure with the MiniArc system is safe and highly effective for SUI. The procedure can be performed in an ambulatory setup and under local anesthesia, with excellent results and patients' satisfaction.

## References

[1] Minassian VA, Stewart WF, Wood GC (2008). Urinary incontinence in women: variation in prevalence estimates and risk factors. Obstet Gynecol.

[2] Kennelly MJ, Moore R, Nguyen JN, Lukban JC, Siegel S (2010). Prospective evaluation of a single incision sling for stress urinary incontinence. J Urol.

[3] Labrie J, Berghmans BL, Fischer K, Milani AL, van der Wijk I, Smalbraak DJ (2013). Surgery versus physiotherapy for stress urinary incontinence. N Engl J Med.

[4] Ulmsten U, Petros P (1995). Intravaginal slingplasty (IVS): an ambulatory surgical procedure for treatment of female urinary incontinence. Scand J Urol Nephrol.

[5] Cox A, Herschorn S, Lee L (2013). Surgical management of female SUI: is there a gold standard?. Nat Rev Urol.

[6] Deng DY, Rutman M, Raz S, Rodriguez LV (2007). Presentation and management of major complications of midurethral slings: Are complications under-reported?. Neurourol Urodyn.

[7] Abdel-Fattah M, Ford JA, Lim CP, Madhuvrata P (2011). Single-incision mini-slings versus standard midurethral slings in surgical management of female stress urinary incontinence: a meta-analysis of effectiveness and complications. Eur Urol.

[8] Wang YJ, Li FP, Wang Q, Yang S, Cai XG, Chen YH (2011). Comparison of three mid-urethral tension-free tapes (TVT, TVT-O, and TVT-Secur) in the treatment of female stress urinary incontinence: 1-year follow-up. Int Urogynecol J.

[9] Moore RD, Mitchell GK, Miklos JR (2009). Single-center retrospective study of the technique, safety, and 12-month efficacy of the MiniArc™ single-incision sling: a new minimally invasive procedure for treatment of female SUI. Surg Technol Int.

[10] Natale F, Dati S, La Penna C, Rombolà P, Cappello S, Piccione E (2014). Single incision sling (Ajust™) for the treatment of female stress urinary incontinence: 2-year follow-up. Eur J Obstet Gynecol Reprod Biol.

[11] Schellart RP, Oude Rengerink K, Van der Aa F, Lucot JP, Kimpe B, de Ridder DJ (2014). A randomized comparison of a single-incision midurethral sling and a transobturator midurethral sling in women with stress urinary incontinence: results of 12-mo follow-up. Eur Urol.

[12] Jiménez-Calvo J, Montesino-Semper M, Hualde-Alfaro A, Torres-Varas L, Sotil-Arrieta A, Raigoso-Ortega O (2015). Stress urinary incontinence surgery with sling MiniArc: a 4-year results. Actas Urol Esp.

[13] De Ridder D, Berkers J, Deprest J, Verguts J, Ost D, Hamid D (2010). Single incision mini-sling versus a transobutaror sling: a comparative study on MiniArc and Monarc slings. Int Urogynecol J.

[14] Oliveira R, Botelho F, Silva P, Resende A, Silva C, Dinis P (2011). Exploratory study assessing efficacy and complications of TVT-O, TVT-Secur, and Mini-Arc: results at 12-month follow-up. Eur Urol.

[15] Moreno K, Montesino M (2007). Economic impact of tension-free vaginal tape surgery for urinary incontinence in an ambulatory regimen compared with hospital admission: a study of the minimization of costs of a public hospital in Spain. Scand J Urol Nephrol.

[16] Presthus JB, Van Drie D, Graham C (2012). MiniArc single-incision sling in the office setting. J Minim Invasive Gynecol.

[17] Jain P, Jirschele K, Botros SM, Latthe PM (2011). Effectiveness of midurethral slings in mixed urinary incontinence: a systematic review and meta-analysis. Int Urogynecol J.

[18] Lee JK, Dwyer PL, Rosamilia A, Lim YN, Polyakov A, Stav K (2011). Persistence of urgency and urge urinary incontinence in women with mixed urinary symptoms after midurethral slings: a multivariate analysis. BJOG.

[19] Meschia M, Barbacini P, Pifarotti P (2008). Multicenter prospective trial of TVT Secur for the treatment of primary stress incontinence. Int Urogyncolog J.

[20] Angleitner-Flotzinger J, Aigmueller T (2014). Mid-term follow-up of the TVT-Secur midurethral sling for primary stress incontinence. Eur J Obstet Gynecol Reprod Biol.

[21] Mostafa A, Lim CP, Hopper L, Madhuvrata P, Abdel-Fattah M (2014). Single-incision mini-slings versus standard midurethral slings in surgical management of female stress urinary incontinence: an updated systematic review and meta-analysis of effectiveness and complications. Eur Urol.

[22] Hunskaar S, Lose G, Sykes D, Voss S (2004). The prevalence of urinary incontinence in women in four European countries. BJU Int.

[23] Nager CW, Brubaker L, Litman HJ, Zyczynski HM, Varner RE, Amundsen C (2012). A randomized trial of urodynamic testing before stress-incontinence surgery. N Engl J Med.

[24] Rachaneni S, Latthe P (2015). Does preoperative urodynamics improve outcomes for women undergoing surgery for stress urinary incontinence? A systematic review and meta-analysis. BJOG.

